# The effect of early coasting on blastocyst development and outcome following blastocyst transfer in IVF/ICSI programme

**DOI:** 10.5935/1518-0557.20180053

**Published:** 2018

**Authors:** Chandra Kailasam, Heather Griffith, Paul Wilson, Uma Gordon

**Affiliations:** 1 Bristol Centre for Reproductive Medicine, Bristol, United Kingdom.

**Keywords:** coasting, blastocyst, live birth, IVF, ovarian hyperstimulation syndrome

## Abstract

**Objective:**

Coasting is a well-known strategy to decrease severity of Ovarian Hyperstimulation Syndrome (OHSS). The purpose of this study is to assess the effect of Coasting on blastocyst development and subsequent clinical outcome following exclusive blastocyst transfer.

**Methods:**

We conducted an observational cohort study of patients having blastocyst transfer following IVF/ICSI treatment. Patients undergoing IVF/ICSI cycles were included in the study. Patients at risk of OHSS were coasted. Outcome following exclusive blastocyst transfer was compared between coasted and non-coasted groups. The main outcome measures were the rate of blastocyst development and live birth rates in coasted and non-coasted cycles. Within coasted cycles, outcome was further analysed based on coasting duration and serum estradiol (E_2_) drop (difference between peak E_2_ and E_2_ on day of HCG).

**Results:**

A total of 166 coasted cycles and 656 non-coasted cycles had blastocyst transfer. Blastocyst development (45.97% vs. 48.6%) and live birth rates (45.18% vs. 43.44%) were not significantly different between coasted and non-coasted cycles. The overall clinical pregnancy (54.21% vs. 49.08%) and implantation rates (43.95% vs. 39.54%) following blastocyst transfer in coasted cycles were not significantly different from those of non-coasted cycles.

**Conclusion:**

Coasting duration up to 6 days and drop in serum E_2_ levels did not compromise blastocyst development, implantation, clinical pregnancy or live birth rates. We conclude that coasting with subsequent blastocyst transfer can be used as an effective strategy in patients at risk of OHSS with no detrimental effects on blastocyst development or live birth outcome.

## INTRODUCTION

Ovarian hyperstimulation syndrome (OHSS) is a potentially life-threatening iatrogenic complication following assisted conception treatment (Roest, 1999; Abramov *et al.,* 1999). It affects approximately 1% of assisted conception cycles in its severe form and a significant proportion of cases may occur in cycles where no risk factors are identified (Delvigne & Rozenberg, 2002). Apart from cycle cancellation, no other preventive measure is guaranteed to have complete efficacy. Although various methods have been proposed for preventing OHSS or diminishing its severity, a survey by Delvigne & Rozenberg (2001) reported that "coasting" was by far the most popular choice.

Coasting was initially described in 1987 (Rabinovici *et al*., 1987) and was first applied in IVF in 1993 (Sher *et al*., 1993). There are many advantages to using this technique. First, the cycle is not abandoned with its attendant cost and emotional implications, while decreasing the incidence and severity of OHSS (García-Velasco *et al*., 2006). The Cochrane review (D’Angelo *et al*., 2011) concluded that coasting reduces the incidence of moderate to severe OHSS when compared with no coasting.

The method of ‘early’ coasting was first introduced by Egbase *et al.* (2002); the lower limit for coasting initiation was estradiol level of 1500 pg/ml (equivalent to 5506 pmol/l) with a leading follicle of 15 mm and this was beneficial in the prevention of OHSS without compromising treatment outcome.

While there are many publications on coasting, these relate to cleavage stage embryos. Our study is the first to look at coasting in relation to exclusive blastocyst transfer. Blastocyst stage is generally known to give better ART outcomes as compared to cleavage stage embryos (Glujovsky *et al.*, 2012); the Cochrane review of 22 RCTs showed that there is a significant difference in live birth rates in favour of blastocyst transfer (Day 5 to 6) compared to cleavage stage transfer (Day 2 to 3). With assisted conception units now preferentially moving to blastocyst transfers, it is important to assess the effect of coasting on blastocyst development and live birth outcome.

Amongst the uncertainties surrounding coasting are the acceptable limits for duration of coasting and rate of fall of serum E_2_. If coasting is initiated too early, then the follicles may undergo atresia with steep fall in E_2_ levels, whereas late coasting may lead to large cystic follicles (Egbase *et al*., 2002). This paper addresses these uncertainties through an observational study undertaken over a period of 6 years.

The aim of the current study was to assess the effect of coasting on blastocyst development rate and live birth outcome as compared to non-coasted cycles. We further analysed the effect of coasting duration and estradiol drop on the same parameters.

## MATERIAL AND METHODS

This observational cohort study enrolled patients undergoing assisted conception treatment at the Bristol Centre for Reproductive Medicine, Bristol, UK from January 2008 to December 2013. We included all patients less than 40 years of age undergoing IVF/ICSI treatment and meeting our criteria for blastocyst transfer. Patients aged ≥40 years and not meeting criteria for blastocyst culture were excluded from the study. The stimulation protocol and laboratory methods have been described in detail previously (Kailasam *et al.,* 2004). In brief, Norethisterone 5 mg twice daily was administered orally for 7 days from the 19^th^ day of the preceding cycle to reduce the incidence of functional ovarian cysts (Aston *et al*., 1995). Gonadotropin releasing hormone agonist (GnRHa) (Intranasal Buserelin acetate spray, Suprefact; Hoechst, UK) 600 µg daily in divided doses was commenced 2 days after starting Norethisterone and serum E_2_ measured 2 weeks later to confirm ovarian suppression. If the E_2_ level was ≤200 pmol/l, gonadotropins (Menogon/Menopur, Ferring, UK or Gonal-F, Serono, UK according to patient preference) were commenced. The initial dose of follicle stimulating hormone (FSH) was 150 IU in patients aged <35 years and 300 IU in patients ≥35 years or varied according to previous ovarian response or risk factors such as polycystic ovarian syndrome.

Transvaginal ultrasonography was used to monitor follicular growth, starting on stimulation day 8 and repeated as necessary. If during follicular tracking more than 20 follicles were noted, serum E_2_ was estimated. When the lead 3 follicles were ≥17mm diameter, Ovitrelle 250µg (equivalent to 6500IU HCG, Merck Serono UK) was administered and transvaginal ultrasound guided oocyte retrieval undertaken 36 hours later. In all of the study treatment cycles, embryos were cultured on to the blastocyst stage and up to two blastocysts transferred. Cyclogest pessaries (Actavis, UK Ltd) at a dose of 400 mg twice daily for two weeks provided luteal phase support. OHSS was graded as per the RCOG Guidelines (2006). All the patients gave written informed consent for fertility treatment and data analysis. Ethical approval was not needed as the study involved analysis of data routinely recorded during fertility treatment.

The outcome following blastocyst transfer was compared between coasted and non-coasted cycles in terms of blastocyst development and clinical outcome i.e. live birth rate, implantation rate and overall pregnancy rate. The clinical pregnancy rate was further analysed based on age, i.e., 35 years and ≥35 years. Clinical pregnancy and implantation rates were further analysed based on the number of blastocysts transferred, i.e., 1 vs. 2 blastocysts transferred. Within the coasted group, the effect of duration of coasting and percentage drop in estradiol (defined as the drop in estradiol from the peak E_2_ level to the E_2_ level on the day of HCG administration) was assessed. Coasted patients were divided into 3 groups based on coasting duration, i.e., 1-2 days, 3-4 days and 4-6 days. The effect of percentage drop in E_2_ was assessed by dividing the patients into 4 groups, i.e., no drop, E_2_ drop 1-30%, E_2_ drop 31-60% and E_2_ drop >60%. The "no drop" estradiol group referred to those cycles wherein the estradiol levels plateaued or rose during coasting. Informed consent was obtained from all individual participants included in the study. Ethical approval was not needed as the study only involved statistical analysis of data routinely recorded during treatment.

### Coasting protocol

Our methodology involved "early coasting," i.e., initiation of coasting when the serum E_2_ level was at least 6000pmol/l and the leading 3 follicles ≥15mm. Subsequently, serum E_2_ level was estimated daily along with scan monitoring as required. Once the lead 3 follicles were ≥17mm, Ovitrelle 250µg (equivalent to 6500 IU HCG, Merck Serono UK) was administered provided the E_2_ levels were ≤12,000pmol/l. Cycles were cancelled if the duration of coasting was more than 6 days or if the serum estradiol levels dropped by more than 65% over 24 hours. In cancelled cycles, GnRH agonist was continued for a further 7 days with the addition of Norethisterone 5 mg orally twice a day. The couples were also advised to avoid sexual intercourse until the next menses.

### Blastocyst culture

Embryos were cultured to the blastocyst stage to aid selection for embryo transfer. Embryo culture was carried out in sequential media manufactured by Origio. The first culture medium (ISM1) is utilised from point of egg collection until 44-52 hours post insemination, at which time the embryos are transferred into the second culture medium (BlastAssist).

Embryos were cultured to blastocyst stage provided they met certain criteria; embryos were assessed each morning from day 2 after egg collection, and if the patient had embryos of good quality and appropriate cell number, more than that required for embryo transfer, the embryos were kept in culture for a further day. Thereafter, if the best embryos for transfer were still not apparent by day 4, they were cultured to the blastocyst stage. The vast majority of blastocyst transfers were on day 5, with only an occasional day 6 transfer; blastocysts were graded according to criteria (Schoolcraft *et al*., 1999).

All patients under the age of 38 years were eligible for elective single blastocyst transfer depending on their previous treatment history and quality of the blastocysts. If a blastocyst of good quality was available (expansion 3 or greater with inner cell mass and trophectoderm grading of BB or better) in the first or second fresh cycle, then a single blastocyst was transferred. If the blastocysts were of poor quality or at an earlier developmental stage, patients were offered the option of a second blastocyst for transfer. The risk of twin pregnancy was strongly emphasised with two blastocyst transfers. Patients aged 38 years or over, and/or those with two failed fresh cycles of treatment, were deemed eligible for a double blastocyst transfer regardless of blastocyst quality.000

### Definitions used

Blastocyst development rate was defined as the number of normally fertilized embryos (2pn) that developed to blastocysts. Implantation rates were defined as the proportion of transferred blastocysts resulting in a gestational sac with foetal heart pulsations. Clinical pregnancy rate was defined by the presence of foetal heartbeat on transvaginal ultrasound scan.

### Assay

Serum estradiol (E_2_) was assayed using a radioimmunoassay (Delfia, Wallac, UK). The inter-assay coefficient of variation was less than 10% over the operating range of the assay and when necessary samples were diluted prior to assay.

### Statistics

Statistical analysis was done using Chi Square tests and z tests where appropriate. A *p* value of <0.05 was considered significant.

## RESULTS

A total of 166 coasted cycles and 656 non-coasted cycles had successful blastocyst culture and transfer.

### Demographic data

The mean patient age was similar between the two groups. The mean number of eggs collected in the coasted group was significantly higher as compared to the non-coasted group (*p*<0.05). Male factor was the commonest cause of infertility in both groups (77.7% in coasted group vs. 80.9% in the non-coasted group). The diagnosis of PCOS was 30.7% in the coasted and 21.2% in the non-coasted group. The proportion of primary infertility was 69% and 73% in the coasted and non-coasted groups respectively, whereas the mean duration of infertility was 2.98 years and 3.14 years in the coasted and non-coasted groups, respectively; the difference was not statistically significant.

### Clinical outcome following blastocyst transfer

Table 1 outlines the clinical outcome in coasted and non-coasted cycles. Blastocyst formation and clinical outcomes (implantation, overall clinical pregnancy and live birth rates) following blastocyst transfer in coasted cycles were not significantly different from those of non-coasted cycles. The clinical pregnancy rates based on age (<35 and ≥35 years) were not significant between the coasted and non-coasted cycles. Similarly, the clinical pregnancy rate was not significantly different wherein patients had a single blastocyst transfer. However, where patients had a double blastocyst transfer the difference in implantation and clinical pregnancy rates between the coasted and non-coasted groups was statistically significant.

**Table 1:** Clinical outcome of blastocyst transfer in coasted vs. non-coasted cycles.

	Blastocyst coasting	Blastocyst non coasting	*p* value
Number of cycles	n=166	n=656	
Eggs collected*	14.81±4.65	12.01±5.05	<0.05
Blastocyst formation rate^¥^	45.97% 669/1455	48.6% 2436/5012	NS
Implantation rate	43.95% 109/248	39.54% 369/933	NS
Implantation rate after 1 blastocyst transfer	55.95% 47/84	54.35% 206/379	NS
Implantation rate after 2 blastocyst transfer	37.81% 62/164	29.42% 163/554	0.04
Clinical Pregnancy rate	54.21% 90/166	49.08% 322/656	NS
Clinical Pregnancy rate <35 years	51.35% 57/111	51.04% 220/431	NS
Clinical Pregnancy rate ≥35 years	55.38% 36/65	45.33% 102/225	NS
Clinical Pregnancy rate 1 blastocyst transfer	53.57% 45/84	53.82% 204/379	NS
Clinical Pregnancy rate 2 blastocyst transfer	54.88% 45/82	42.59% 118/277	0.05
Live Birth Rate	45.18% 75/166	43.44% 285/656	NS
Multiple pregnancy rate	11.44% 19/166	7.01% 46/656	NS

* =mean ± std dev

¥ = number of blastocysts / number of 2pn embryos

NS =not significant; *p*<0.05 significant

### Blastocyst transfer outcome by duration of coasting

Table 2 outlines the effect of coasting duration for up to 6 days on blastocyst transfer outcome. No impact on blastocyst development or clinical outcome was noted.

**Table 2:** Effect of coasting duration on blastocyst formation rate & clinical outcome.

	Coasting Duration			
	1–2 days n=69	3–4 days n=62	5-6 days n=35	*p* value
Blastocyst formation rate	44.23% 288/651	46.94% 246/524	46.42% 130/280	NS
Implantation rate	37.86% 39/103	50.56% 45/89	44.64 25/56	NS
Clinical Pregnancy rate	46.37% 32/69	59.67% 37/62	60% 21/35	NS
Live birth rate	39.13% 27/69	51.61% 32/62	45.71% 16/35	NS

NS =not significant

### **Blastocyst transfer outcome by E**_2_ **drop**

Table 3 shows that E_2_ drop had no significant impact on blastocyst development or clinical outcome following blastocyst transfer.

**Table 3:** Effect of serum estradiol drop on blastocyst formation rate & clinical outcome.

	Estradiol (E_2_) drop %				
	No E_2_ drop n=70	E_2_ drop 1–30% n=29	E_2_ drop 31 – 60% n=39	E_2_ drop > 60% n=28	*p* value
Blastocyst formation rate	44.73% 293/655	47.08% 129/274	49.37% 159/322	43.13% 88/204	NS
Implantation rate	39.21%% 40/102	52.38% 22/42	53.23% 33/62	33.33% 14/42	NS
Clinical Pregnancy rate	47.14% 33/70	62.06% 18/29	64.10% 25/39	50% 14/28	NS
Live birth rate	40% 28/70	58.62% 17/29	51.28% 20/39	35.71% 10/28	NS

The multiple pregnancy rate was 11.44% in the coasted group and 7.01% (46/656) in the non-coasted group. There was no case of severe OHSS in the coasted group whereas the incidence of severe OHSS was less than 1% in the non-coasted group.

## DISCUSSION

### Main findings

Coasting, a strategy to decrease OHSS in patients who overrespond to ovarian stimulation, does not compromise blastocyst development or live birth rates following blastocyst transfer and yields outcomes similar to non-coasted cycles. Coasting duration up to 6 days and estradiol drop within the confines of our study had no impact on blastocyst development or live birth outcome following blastocyst transfer.

### Strengths and Limitations

To our knowledge, this is the first study that looked at coasted cycle outcome with blastocyst transfer exclusively. Early coasting was used as an interventional strategy in patients who overresponded to ovarian stimulation in IVF/ICSI cycles.

Our study used the long protocol GnRH-a regime for all cycles with coasting as a preventive strategy to decrease risk of OHSS in patients showing over-response. However, there are other tools to decrease risk of OHSS. Use of GnRH antagonists, along with use of agonist trigger for final follicular maturation, is increasingly being recommended in high-risk patients.

### Interpretation

With early coasting, the size of the leading follicle is important since follicles 15mm or larger can continue to grow for a few days even in the absence of further gonadotropin support (Levinsohn‐Tavor *et al*., 2003). This also compares to spontaneous ovulatory cycle data that follicular diameter of 15 mm is probably the critical follicular size prerequisite for LH surge (Cahill et al., 1998). Hence an estradiol level of 1500 pg/ml with 15mm leading follicle was considered the minimum level at which coasting could be safely initiated.

In our study, we initiated our early coasting protocol with the leading follicle at 15 mm and estradiol level of at least 6000 pmol/L. Estradiol levels generally continue to rise for a few days in overresponders (more than 20 follicles) before a fall in serum E_2_. When serum E_2_ fell to 12,000pmol/L (safe level), HCG was administered. In a survey undertaken by Delvigne & Rozenberg (2001), coasting was initiated at varying serum E2 levels. Most physicians who used coasting selected an E_2_ concentration of 3,000pg/ml, equivalent to 11,000pmol/l, as the upper limit to administer hCG. As per Egbase *et al.* (2002), coasting at an earlier stage (leading follicle of 15mm and serum E_2_ levels >1500 pg/ml (5506pmol/L) in patients with excessive ovarian follicular response is consistent with good embryological and clinical outcome.

The number of oocytes collected in our study was significantly higher in the coasted group as compared to the non-coasted group (*p*<0.05). Other authors had reported this finding (Talebi Chahvar *et al*., 2014), although Mansour *et al*. (2005) reported a reduction in the number of oocytes with prolonged coasting. Even though a higher number of eggs was retrieved from coasted cycles in our study, this did not have an impact on the blastocyst formation rate, which was similar between coasted and non-coasted cycles.

Our policy is to aim for blastocyst transfer in all patients who meet the blastocyst culture criteria, in view of the expected better outcome compared to cleavage stage embryos (Glujovsky *et al*., 2012). A retrospective case-control study (Talebi Chahvar *et al*., 2014) also looked at blastocyst development rate and outcomes; however, their live birth outcome comparison related to embryos of all stages and not exclusively blastocyst transfers as in our study. Their methodology also involved initiation of coasting at serum E_2_ level >13,000 pmol/L, unlike early coasting as applied in our study.

One of the controversial aspects of coasting - and still an issue over which there is no consensus - relates to the maximum duration of coasting that can be undertaken without compromising outcome. Our study findings showed no deleterious effect on blastocyst development or live birth rate up to 6 days of coasting. Other studies, involving embryo stage transfer, have shown a reduction in the number and quality of the oocytes with reduced endometrial receptivity when coasting lasted for ≥4 days (García-Velasco *et al*., 2006). A large retrospective analysis (Mansour *et al*., 2005) of 1,223 cycles coasted for a maximum of 7 days reported that fertilization rate was not different between groups, although the embryo implantation and clinical pregnancy rates were significantly reduced in patients coasted for >3 days.

In contrast, Nardo *et al*. (2006) reported that oocyte maturity, fertilization and embryo cleavage rates were similar between patients coasted 1-3 days (n=57) vs. ≥4days (n=21), while women coasted for ≥4days had a decrease in implantation rate compared to individuals coasted for 1-3 days; there were no significant differences in the pregnancy/embryo transfer, live birth or cancellation rates between groups. While there is concern that prolonged coasting can exert a negative impact on IVF cycle outcome (Moreno *et al*., 2004), a retrospective analysis (Abdalla & Nicopoullous, 2010) of 1068 coasted cycles over 13 years reported no difference in live birth rates with up to eight days coasting; although cycle numbers beyond 6 days of coasting were small (n=34), the live birth rate per cycle started was 28.6% with up to 8 days of coasting; no live births were reported with the 5 cycles that went to 9 days of coasting. In our study, treatment was cancelled if the duration of coasting was more than 6 days or if the serum estradiol levels dropped by more than 65% over 24 hours due to potential concerns on egg quality.

In our study, the clinical pregnancy rates were similar wherein patients had a single blastocyst transfer. However, where patients had a double blastocyst transfer, the difference in implantation and clinical pregnancy rates was statistically significant between coasted and non-coasted groups. While there was no obvious reason for this finding, it was reassuring that coasting did not compromise the outcome.

Coasting also involves monitoring by daily estimation of serum E_2_ levels. Even though a high E_2_ level is recognised as a risk factor, it is poorly predictive of OHSS (Morris *et al*., 1995; Orvieto, 2003). There is however a general concern that an excessive drop in E_2_ levels during the coasting period may compromise outcome and our study assessed the impact of change in serum estradiol levels. Ours is the first study that looked at this aspect in exclusive blastocyst transfers with coasting and showed that there is no significant decline in blastocyst formation, implantation, clinical pregnancy rates or live birth rates when comparing the degree of E_2_ drop from peak E_2_ level to the E_2_ level on day of HCG. A large retrospective study (Abdalla & Nicopoullous, 2010) showed no difference in live birth rates based on the E_2_ levels on the day of hCG or E_2_ drop. In a retrospective analysis (Kovács *et al*., 2006) (n=39), pregnancy rates were comparable when groups were assessed based on the percentage change in E_2_ from peak estradiol level (<25%, 25%-50%, >50%). A retrospective review of 346 patients (Ulun *et al*., 2004) reported that when E_2_ decrement during coasting period was evaluated in coasted cycles, no correlation was found between the change in E_2_ and either fertilization rates or pregnancy rates. Similarly in a literature review of 131 coasted cycles, the decrease in E_2_ levels from the initial E_2_ level or from the highest E_2_ level observed to the day of hCG administration had no predictive value in either IVF patients or egg donors (García-Velasco *et al*., 2006).

The Cochrane group concluded that compared to the long GnRH agonist protocol, GnRH antagonists significantly reduced the incidence of OHSS with no evidence of a difference in live birth rates (Al-Inany *et al.*, 2011). We are increasingly using the antagonist protocol for patients at risk of OHSS. The same criteria for coasting can be applied in GnRH agonist and antagonist protocols, with similar laboratory performance and cycle outcome (Farhi *et al*., 2009). Secondly, advances in vitrification technology to freeze embryos have improved the outcome of frozen embryo transfers approaching that of fresh embryo transfers, thereby eliminating the risk of late OHSS in high risk patients (Maheshwari & Bhattacharya, 2013). However, while initial results may support replacement all fresh IVF/ICSI cycles with frozen embryo transfer cycles as a safer and equally effective strategy, robust evidence from randomized controlled trials is needed if this will be generally applied (Kalampokas *et al*., 2015).

The authors acknowledge that the increasing popularity of antagonist protocols and advances in embryo freezing with deferred embryo transfer has decreased the need for coasting. However, the long agonist protocol regime continues to be used widely, especially in patients wherein there is a low risk of ovarian over-response. Our study provides reassuring evidence, in this group, regarding the effect of coasting on blastocyst development and outcome.

## CONCLUSION

Coasting does not compromise blastocyst development or live birth outcome following blastocyst transfer. Coasting duration up to 6 days and estradiol drop within the confines of our study had no detrimental outcome.
